# Interpretable four-class machine learning prediction of initial I-131 therapy responses in differentiated thyroid cancer

**DOI:** 10.1080/07853890.2026.2729596

**Published:** 2026-09-10

**Authors:** Yangyang Lei, Yi Mao, Rui Zhu, Zhaoxia Luo, Qing Zhang

**Affiliations:** a Department of Nuclear Medicine, The First Affiliated Hospital, Jiangxi Medical College, Nanchang University, Nanchang, China; b Department of Ultrasound Medicine, The First Affiliated Hospital, Jiangxi Medical College, Nanchang University, Nanchang, China

**Keywords:** Differentiated thyroid carcinoma, I-131 therapy, machine learning, four-class prediction, SHAP

## Abstract

**Background:**

Differentiated thyroid carcinoma (DTC) responses to initial post-operative I-131 therapy are heterogeneous. Standard binary models combine incomplete responses into a ‘non-excellent’ category, obscuring transitional states (indeterminate response [IDR] and biochemical incomplete response [BIR]) and outcome-specific drivers. We developed and validated an interpretable four-class machine learning framework to separate these dynamic trajectories and support pre-therapeutic decisions.

**Materials and methods:**

Data from 948 DTC patients were partitioned into training (*n* = 663) and test (*n* = 285) cohorts, with an independent temporal validation cohort (n=150). Four algorithms (Random Forest [RF], eXtreme Gradient Boosting [XGBoost], Light Gradient Boosting Machine [LightGBM] and Multi-Layer Perceptron [MLP]) were evaluated to predict excellent response (ER), IDR, BIR and structural incomplete response (SIR). Performance was assessed *via* area under the receiver operating characteristic curve (AUC), calibration curves and decision curve analysis (DCA). Shapley Additive exPlanations (SHAP) and multivariable logistic regression quantified category-specific drivers.

**Results:**

Random forest achieved a micro-average AUC of 0.894 (95% CI: 0.842–0.901) in the test cohort and 0.885 (95% CI: 0.832–0.912) in validation. SHAP analysis revealed that pre-treatment stimulated thyroglobulin (pre-sTg) and stimulated thyroglobulin to thyroid-stimulating hormone ratio (LOG(sTg/TSH)) dominated ER, IDR and BIR predictions (pre-sTg peak weight: 31.9% in BIR), whereas diagnostic whole-body scintigraphy (Dx-WBS) was definitive for SIR (45.1% weight). Distant metastasis on Dx-WBS dramatically elevated SIR risk (OR: 171.89); pre-sTg ≥ 10 ng/mL significantly increased both SIR (OR: 7.18) and BIR (OR: 5.48) risks.

**Conclusions:**

This four-class framework separates biochemical and structural drivers of post-I-131 responses, providing a practical risk-stratification tool for personalized management before therapy.

## KEY MESSAGES

1. Traditional binary models obscure the divergent driving mechanisms between biochemical and structural incomplete responses in differentiated thyroid carcinoma after initial I-131 therapy.
2. An interpretable four-class machine learning framework accurately segregates response categories and reveals outcome-specific predictive drivers using SHAP analysis.
3. Pre-treatment stimulated thyroglobulin dominates biochemical persistence, whereas diagnostic whole-body scintigraphy findings serve as the definitive engine for structural progression.

## Introduction

Differentiated thyroid carcinoma (DTC) represents the most prevalent malignant neoplasm of the endocrine system, with a global incidence that exhibits a consistent upward trend [1]. Post-operative I-131 therapy reduces recurrence risk in intermediate/high-risk patients, but responses vary markedly. The range of outcomes encompasses excellent response (ER), biochemical incomplete response (BIR), indeterminate response (IDR) and structural incomplete response (SIR) [2]. Crucially, these response categories are not strictly static compartments but rather represent a continuous clinical-biological spectrum [3]. Within this framework, IDR acts as a highly volatile and heterogeneous transitional zone bridging excellent outcomes and covert progression, making its early discrimination both clinically paramount and inherently challenging [4]. The 5-year recurrence rate for SIR patients exceeds 60%, while it is less than 5% for ER patients [5]. Pre-treatment prediction is, therefore, imperative for the implementation of a personalized management strategy.

Traditional efficacy prediction relies heavily on static pathological features such as TNM staging and American Thyroid Association (ATA) recurrence risk stratification, which lacks sensitivity for dynamic treatment response [3,6]. Recently, machine learning (ML) has been widely adopted in medical prediction due to its strength in integrating multi-dimensional data and uncovering complex non-linear relationships [7,8]. However, extant studies were encumbered by significant limitations. Firstly, conventional binary models combine distinct incomplete responses into a single ‘non-ER’ lump, effectively obfuscating the divergent driving mechanisms behind purely biochemical persistence (BIR) versus macrometastatic structural progression (SIR) [9]. Furthermore, by replacing the nuanced spectrum with a rigid dichotomy, such models completely erase the transitional probability gradients of IDR, preventing clinicians from identifying patients hovering at the crucial crossroad of disease evolution [10]. Early intervention can reduce progression risk by more than 50% since BIR precedes structural lesions by 6–12 months, yet binary models delay such tailored insights [10,11]. Secondly, most models were characterized by a lack of interpretability. This property of ‘black box’ models hinders their clinical adoption, since it prevents the discernment of outcome-specific drivers [7]. However, the paucity of studies that have examined differential mechanisms across four distinct outcomes has hindered the translation of findings into clinical practice.

In order to address the aforementioned gaps, an interpretable four-class machine learning model predicting post-I-131 therapy outcomes in DTC was developed. A comparison of four algorithms – including Random Forest (RF), eXtreme Gradient Boosting (XGBoost), Multi-Layer Perceptron (MLP) and Light Gradient Boosting Machine (LightGBM) – with independent validation was conducted, and Shapley Additive exPlanations (SHAP) analysis was utilized to quantify feature importance across response categories.

## Methods

### Study population and data partitioning

DTC patients who underwent total thyroidectomy followed by initial I-131 therapy were enrolled betweenOctober 2020 and March 2024 for the retrospective development cohort (*n* = 948; training set: *n* = 663, test set: *n* = 285). An independent temporal validation cohort (*n* = 150) was recruited between October 2024 and March 2025. Patient management and follow-up protocols remained strictly consistent across both timeframes. Electronic medical records for both the development cohort and the independent temporal validation cohort were accessed and extracted between 18 April 2026 and 5 June 2026, following formal ethical approval. Inclusion criteria required histologically confirmed DTC and ≥ 6 months of follow-up with documented response assessments. Patients with incomplete data or concurrent malignancies were excluded. This study complied with the STROBE guidelines.

### Data preprocessing and statistical analysis

Categorical variables were analysed using the chi-square test. Data normality was evaluated *via* the Shapiro-Wilk test. Continuous variables were expressed as mean ± SD (normal distribution) or median with interquartile range (IQR; non-normal distribution). Group comparisons utilized one-way ANOVA (homogeneous variance *via* Levene’s test) or the Kruskal-Wallis H test. Post hoc pairwise comparisons were performed using Tukey’s HSD or Dunn’s test with Bonferroni correction, setting significance at *p* < 0.0083 (0.05/6). Violin plots visualized the predicted risk score distributions. To optimize algorithmic performance, the pre-ablation sTg/TSH ratio was logarithmically transformed into LOG(sTg/TSH).

### Feature selection and dimensionality reduction

Feature selection was restricted to the training set. Variables with *p* < 0.05 in univariate analysis were evaluated for multicollinearity using the variance inflation factor (VIF). Eliminating features with VIF > 10 yielded 13 core predictors: pre-treatment stimulated thyroglobulin (pre-sTg), LOG(sTg/TSH) ratio, pre-treatment anti-thyroglobulin antibody (pre-TgAb) status, N stage, diagnostic whole-body scintigraphy (Dx-WBS) findings (pre-therapeutic dose: 222 MBq; criteria in Table 1), maximum diameter of metastatic lymph nodes (MDMLN), T stage, lesion number, thyroid ultrasound, chest CT, M stage, clinical stage and pathological type. TNM staging and treatment responses (ER, IDR, BIR, SIR) followed the AJCC 8th edition and 2025 ATA guidelines, respectively.

**Table 1.** Distribution of baseline characteristics of the study population.

Clinical data		Total (*N* = 948)	ER	IDR	BIR	SIR
Efficacy (%)	ER	372 (39.2)				
	IDR	292 (30.8)				
	BIR	165 (17.4)				
	SIR	119 (12.6)				
Gender	Male	293 (30.9)	106 (28.5)	93 (31.8)	53 (32.1)	41 (34.4)
	Female	655 (69.1)	266 (71.5)	199 (68.2)	112 (67.9)	78 (65.6)
^I-131^ Therapy interval	<3 months	641 (67.6)	270 (72.8)	193 (65.7)	102 (61.8)	76 (63.9)
	>3 months	307 (32.4)	102 (27.2)	99 (34.3)	63 (38.2)	43 (36.1)
Tumor unilateral/bilateral	Unilateral	420 (44.3)	159 (42.9)	111 (38.5)	101 (60.1)	49 (42.9)
	Bilateral	528 (55.7)	213 (57.1)	181 (61.5)	64 (39.9)	70 (57.1)
Extrathyroidal invasion	Yes	422 (44.4)	160 (43.0)	121 (41.4)	80 (48.5)	61 (51.3)
	No	526 (55.6)	212 (57.0)	171 (58.6)	85 (51.5)	58 (48.7)
Tumor focality	Single	402 (42.4)	177 (47.6)	113 (38.7)	67 (40.6)	45 (37.8)
	Multiple	546 (57.6)	195 (52.4)	179 (61.3)	98 (59.4)	74 (62.2)
Lateral cervical LN metastasis	Yes	536 (55.3)	155 (41.7)	184 (63.0)	112 (67.9)	85 (71.4)
	No	412 (44.7)	217 (58.3)	108 (37.0)	53 (32.1)	34 (28.6)
Histology	Classic	849 (89.6)	332 (89.2)	268 (91.7)	156 (94.5)	93 (78.1)
	Non-classic	77 (8.1)	35 (9.5)	24 (8.3)	6 (3.6)	12 (10.1)
	Follicular carcinoma	22 (2.3)	5 (1.3)	0 (0)	3 (1.9)	14 (11.8)
TgAb	Negative	800 (84.4)	347 (93.3)	216 (74.0)	138 (83.6)	99 (83.2)
	Positive	148 (15.6)	25 (6.7)	76 (26.0)	27 (16.4)	20 (16.8)
T stage	TX	41 (4.3)	11 (3.0)	8 (2.7)	6 (3.6)	16 (13.4)
	T1a	155 (16.5)	80 (21.5)	52 (17.8)	16 (9.6)	7 (5.9)
	T1b	178 (18.8)	75 (20.2)	62 (21.2)	28 (17.0)	13 (10.9)
	T2	128 (13.5)	43 (11.5)	36 (12.3)	30 (18.1)	19 (16.0)
	T3a	25 (2.6)	3 (0.8)	13 (4.5)	5 (3.0)	4 (3.4)
	T3b	294 (31.0)	110 (29.6)	93 (31.8)	49 (30.0)	42 (35.3)
	T4a	120 (12.7)	48 (12.9)	26 (8.9)	30(18.1)	16 (13.4)
	T4b	7 (0.7)	2 (0.5)	2 (0.8)	1 (0.6)	2 (1.7)
N Stage	NX	6 (0.6)	0 (0)	2 (0.7)	1 (0.6)	3 (2.5)
	N0	66 (7.0)	40 (10.8)	11 (3.8)	7 (4.2)	8 (6.7)
	N1a	323 (34.1)	170 (45.7)	87 (29.8)	42 (25.5)	24 (20.2)
	N1b	553 (58.3)	162 (43.5)	192 (65.7)	115 (69.7)	84 (70.6)
M Stage	MX	9 (0.9)	0 (0)	0 (0)	4 (2.4)	5 (4.2)
	M0	911 (96.1)	372(100)	290(99.3)	160 (97.0)	89 (74.8)
	M1	28 (3.0)	0 (0)	2 (0.7)	1 (0.6)	25 (21.0)
Clinical Stage	I	791 (83.4)	319 (85.8)	262 (89.7)	142 (86.1)	68 (57.1)
	II	111 (11.7)	39 (10.5)	25 (8.6)	15 (9.1)	32 (26.9)
	III	29 (3.1)	14 (3.7)	4 (1.4)	8 (4.8)	3 (2.5)
	IVA	3 (0.3)	0 (0)	1 (0.3)	0 (0)	2 (1.7)

**Table:** 

Clinical data		Total (*N* = 948)	ER	IDR	BIR	SIR
	IVB	14 (1.5)	0 (0)	0 (0)	0 (0)	14 (11.8)
Recurrence risk stratification	Low-risk	128 (13.5)	78(21.0)	36(12.3)	10(6.1)	4(3.4)
	Intermediate-risk	566 (59.7)	260(69.9)	206(70.5)	71(43.0)	29(24.4)
	High-risk	254 (26.8)	34 (9.1)	50 (17.2)	84 (50.9)	86 (72.3)
US findings	No metastasis	836 (88.2)	349(93.8)	267 (91.4)	141 (85.5)	79 (66.4)
	LN metastasis	112 (11.8)	23(6.2)	25 (8.6)	24 (14.5)	40 (33.6)
CT findings	No metastasis	916 (96.6)	372 (100.0)	289 (99.0)	165 (100.0)	90 (75.6)
	Metastasis	32 (3.4)	0 (0)	3 (1.0)	0 (0)	29 (24.4)
Dx-WBS Findings	No metastasis	824 (86.9)	364 (97.8)	279 (95.5)	157 (95.2)	24 (20.2)
	LN metastasis	94 (9.9)	8 (2.2)	10 (3.5)	8 (4.8)	68 (57.1)
	DM	30 (3.2)	0 (0)	3 (1.0)	0 (0)	27 (22.7)
Age (years)		40 (31.8, 50)	43 (33.5, 51)	37 (31, 47)	37 (28, 47)	47 (32, 58.5)
Tumor size(cm)		1.6 (1.1,2.5)	1.5 (1.0, 2.0)	1.6 (1.1, 2.2)	1.8 (1.3, 2.5)	2.0 (1.5, 3.1)
MDMLN(cm)		0.9 (0.6,1.1)	0.8 (0.4, 1.0)	0.9 (0.6, 1.1)	1.0 (0.8, 1.2)	1.0 (0.8, 1.4)
pre-TSH (mIU/L)		82.1 (61.1,100)	79.6 (60.6, 100)	82.6 (61.6, 100)	85.0 (62.2, 100)	79.1 (58.1, 100)
pre-sTg (ng/mL)		4.2 (0.6, 15.9)	1.6 (0.3, 4.1)	4.5 (0.3, 10.5)	25.1 (8.9, 58.2)	78.4 (10.5, 406.0)
sTg/TSH ratio		0.05 (0.01, 0.2)	0.02 (0.01, 0.05)	0.06 (0.01, 0.14)	0.3 (0.1, 0.7)	0.9 (0.2, 5.7)
Weight (kg)		61.5 (55,70)	61 (55, 70)	61 (55, 70)	62 (55, 71)	62.5 (54.8, 70)
Height (cm)		160 (157,167)	160 (157, 165)	161 (157,168)	160 (156, 168)	160 (157, 167.5)
BMI (kg/m²)		23.8 (21.5,26.1)	23.9 (21.7, 26.4)	23.8(21.3, 25.7)	24.1 (21.4, 26.3)	23.6 (21.6, 25.6)
I-131 Dose MBq (mCi)		5550 (150)	5550 (150)	5550 (150)	6660 (180)	6660 (180)
		[5550, 6660 (150, 180)]	[5550, 5550 (150,150)]	[5550, 5920 (150, 160)]	[5550, 6660 (150, 180)]	[5920, 7400 (160, 200)]

* Data are presented as n (%) or median (IQR) unless otherwise indicated.

**Abbreviations:** ER, excellent response; IDR, indeterminate response; BIR, biochemical incomplete response; SIR, structural incomplete response; IQR, interquartile range; ATA, American Thyroid Association; pre-TgAb, pre-treatment thyroglobulin antibody; pre-sTg, pre-treatment stimulated thyroglobulin; pre-TSH, pre-treatment thyroid-stimulating hormone; sTg/TSH ratio, stimulated thyroglobulin-to-thyroid-stimulating hormone ratio; Dx-WBS, diagnostic whole-body scintigraphy; MDMLN, maximum diameter of metastatic lymph node; LN, lymph node; DM, distant metastasis; US, ultrasound; CT, computed tomography; BMI, body mass index; TNM, tumour-node-metastasis; MBq, megabecquerel; mCi, millicurie.

**Note:** In accordance with journal guidelines, I-131 therapeutic doses are simultaneously expressed in both Megabecquerels (MBq) as the primary unit and Millicuries (mCi) in parentheses: 5550 MBq (150 mCi), 5920 MBq (160 mCi), 6660 MBq (180 mCi) and 7400 MBq (200 mCi). Diagnostic whole-body scintigraphy (Dx-WBS) was performed with a diagnostic dose of 222 MBq (6 mCi) of I-131 prior to therapeutic administration. **Dx-WBS findings were visually evaluated by two nuclear medicine physicians, with additional SPECT/CT performed for suspicious focal lesions to categorize uptake into Dx-WBS(-), Dx-WBS:LN(+) and Dx-WBS:DM(+). Serum pre-sTg was assessed across all patients, with pre-TgAb status incorporated as an independent covariate to account for potential antibody immunoassay interference during downstream modeling**.

### Construction and training of machine learning models

Four multi-class models (RF, XGBoost, MLP and LightGBM) were constructed using the 13 features and optimized *via* fivefold nested cross-validation on the training set. For XGBoost and LightGBM, multi-class software objectives were applied with strict regularization (max_depth ≤ 6, learning_rate ≤ 0.05, min_child_weight ≥ 5) and early stopping (patience = 10 epochs) to mitigate overfitting.

### Model evaluation and validation

Discrimination was evaluated on the test and temporal validation sets using micro-/macro-average and class-specific one-vs-rest AUCs with 95% CIs. Calibration curves assessed probability accuracy, while decision curve analysis (DCA) determined net clinical benefit. Confusion matrix heatmaps visualized classification accuracy across the four categories.

### SHAP analysis and visualization

The top-performing RF model was interpreted globally and locally using the Python shap library. Beeswarm plots displayed the directional impact of features, and donut charts quantified mean absolute SHAP values to represent the relative feature importance within each response category.

### Forest plot construction

Multivariable logistic regression identified independent predictors for distinct endpoints using binary comparison frameworks (e.g. SIR vs. Others, ER vs. Others). For categorical predictors, baseline categories served as reference (pre-sTg < 1 ng/mL, Dx-WBS(-), N0 and pre-TgAb(-)). Results were visualized as adjusted odds ratios (ORs) and 95% CIs using Python’s forestplot library.

## Results

### Baseline characteristics of the study population

A total of 1,098 DTC patients were evaluated, including a retrospective development cohort (*n* = 948; 663 training and 285 test sets) and an independent temporal validation cohort (*n* = 150). Within the development cohort, the therapeutic response distribution was 39.2% ER (*n* = 372), 30.8% IDR (*n* = 292), 17.4% BIR (*n* = 165) and 12.6% SIR (*n* = 119). Univariate analysis demonstrated significant baseline heterogeneity across these four response categories (*p* < 0.0083, adjusting for multiple pairwise comparisons), establishing a progressive tumour-burden gradient from ER to SIR (Table 1; see Supplementary Table 1 for data set partitioning details).

### Univariate analysis and feature selection

Univariate analysis demonstrated significant baseline heterogeneities across the four response groups for 18 of the 26 evaluated features (*p* < 0.0083) (Table 2). The most pronounced statistical differences were identified in Dx-WBS findings ($\chi^{2}$ = 541.44, *p* < 0.001) and pre-sTg levels (*H* = 276.77, *p* < 0.001). Following multi-collinearity filtering *via* variance inflation factor (VIF) analysis (exclusion threshold: VIF > 10), 13 non-redundant core variables were successfully retained for subsequent machine learning modelling.

**Table 2.** Univariate analysis of clinical features across ER, IDR, BIR and SIR groups.

Feature	*χ²*	*p*	Feature	*Kruskal-H*	*p*
Gender	1.95	0.580	Age	36.42	<0.001
Therapy interval	7.79	0.050	Tumour Size	51.84	<0.001
Tumour unilateral/bilateral	1.75	0.630	MDMLN	26.15	<0.001
Tumour focality	21.11	<0.005	pre-TSH	6.26	0.100
Extrathyroidal invasion	4.70	0.190	pre-sTg	276.77	<0.001
Lateral cervical LN metastasis	57.84	<0.001	sTg/TSH ratio	262.20	<0.001
Histology	61.98	<0.001	Weight	0.48	0.920
pre-TgAb	46.57	<0.001	Height	2.53	0.470
T stage	74.11	<0.001	BMI	1.07	0.780
N stage	72.88	<0.001	I-131 Dose	219.46	<0.001
M stage	180.37	<0.001			
Clinical stage	150.36	<0.001			
Recurrence risk stratification	257.40	<0.001			
US findings	76.94	<0.001			
CT findings	169.90	<0.001			
Dx-WBS findings	541.44	<0.001			

**Abbreviations:** pre-TgAb, pre-treatment thyroglobulin antibody; pre-sTg, pre-treatment stimulated thyroglobulin; pre-TSH, pre-treatment thyroid-stimulating hormone; sTg/TSH ratio, stimulated thyroglobulin-to-thyroid-stimulating hormone ratio; US, ultrasound; CT, computed tomography; Dx-WBS, diagnostic whole-body scintigraphy; MDMLN, maximum diameter of metastatic lymph node; BMI, body mass index; TNM, tumour-node-metastasis; *χ^2^*, chi-square test statistic; H/Kruskal-H, Kruskal-Wallis H test statistic.

### Model performance across data sets

**Figure 1.**
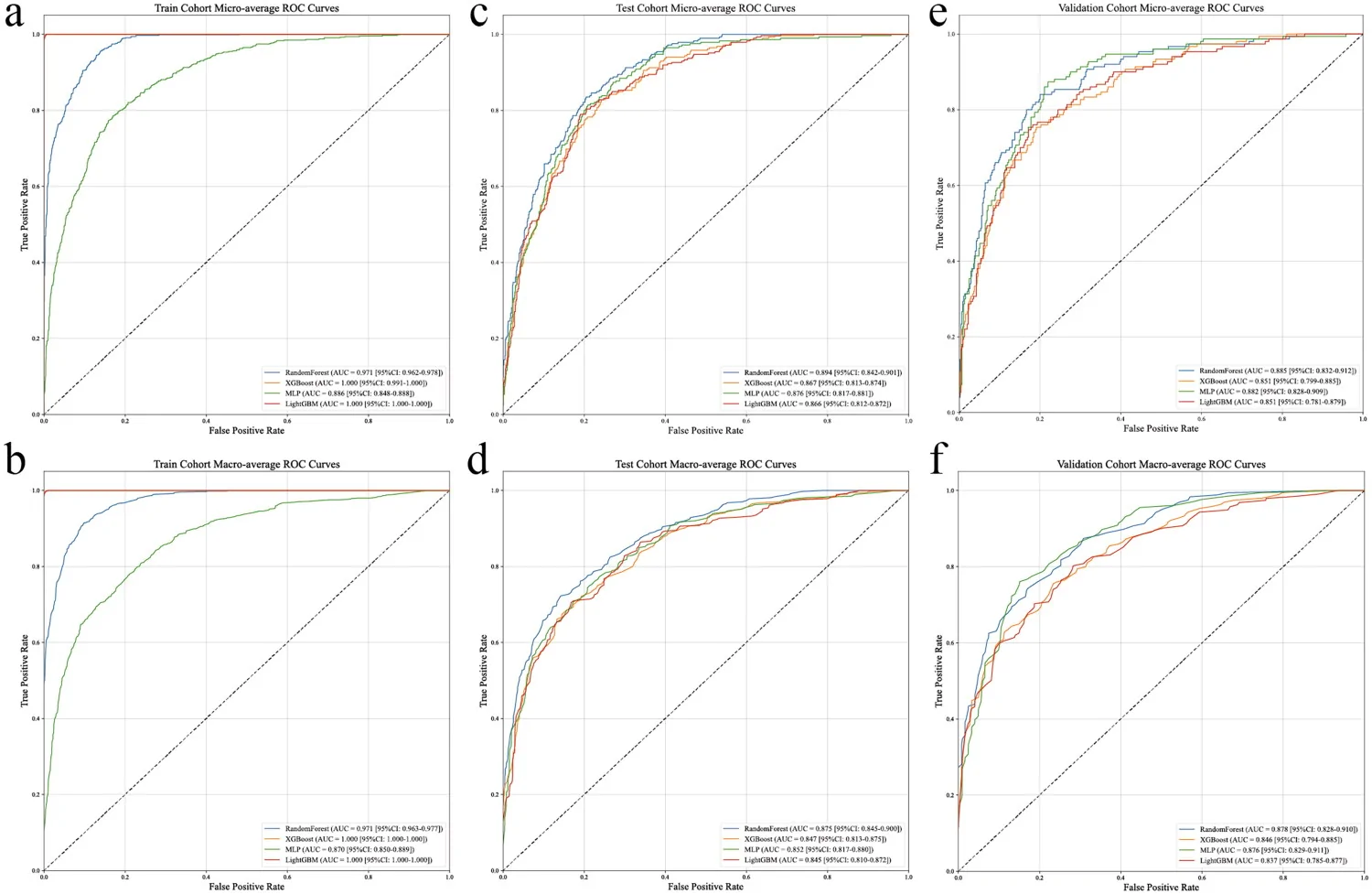
**Performance of four machine learning models across training, test, and temporal validation cohorts.** Micro-average (top row) and macro-average (bottom row) receiver operating characteristic (ROC) curves are evaluated for the RF, XGBoost, LightGBM and MLP algorithms. (a, b) Model discrimination in the training cohort (*n* = 663), where gradient boosting algorithms exhibit mathematical saturation. (c, d) Performance in the randomized test cohort (*n* = 285), establishing the superior generalizability of the RF model. (e, f) Performance in the independent temporal validation cohort (*n* = 150), demonstrating the stability and robustness of the RF framework across non-overlapping populations.

The comparative performance of the four algorithms is illustrated in Figure 1. In the training cohort, XGBoost and LightGBM achieved an AUC of 1.000, indicating mathematical saturation, whereas the RF model exhibited a training AUC of 0.971 (Figure 1a and 1b). However, in the randomized test cohort, the RF model demonstrated superior generalizability with a micro-average AUC of 0.894 (95% CI: 0.842–0.901) and a macro-average AUC of 0.875 (95% CI: 0.845–0.900), while boosting models suffered a substantial performance decline due to over-fitting (Figure 1c and 1d). Crucially, in the independent temporal validation cohort, the RF model sustained optimal stability, achieving a micro-average AUC of 0.885 (95% CI: 0.832–0.912) and a macro-average AUC of 0.878 (95% CI: 0.828–0.910), confirming its robust external generalizability (Figure 1e and 1f). **Detailed class-specific metrics, including sensitivity, specificity, accuracy and F1 scores across all cohorts, are provided in** Supplementary Table 2.

### Calibration curve and decision curve analysis of models

**Figure 2.**
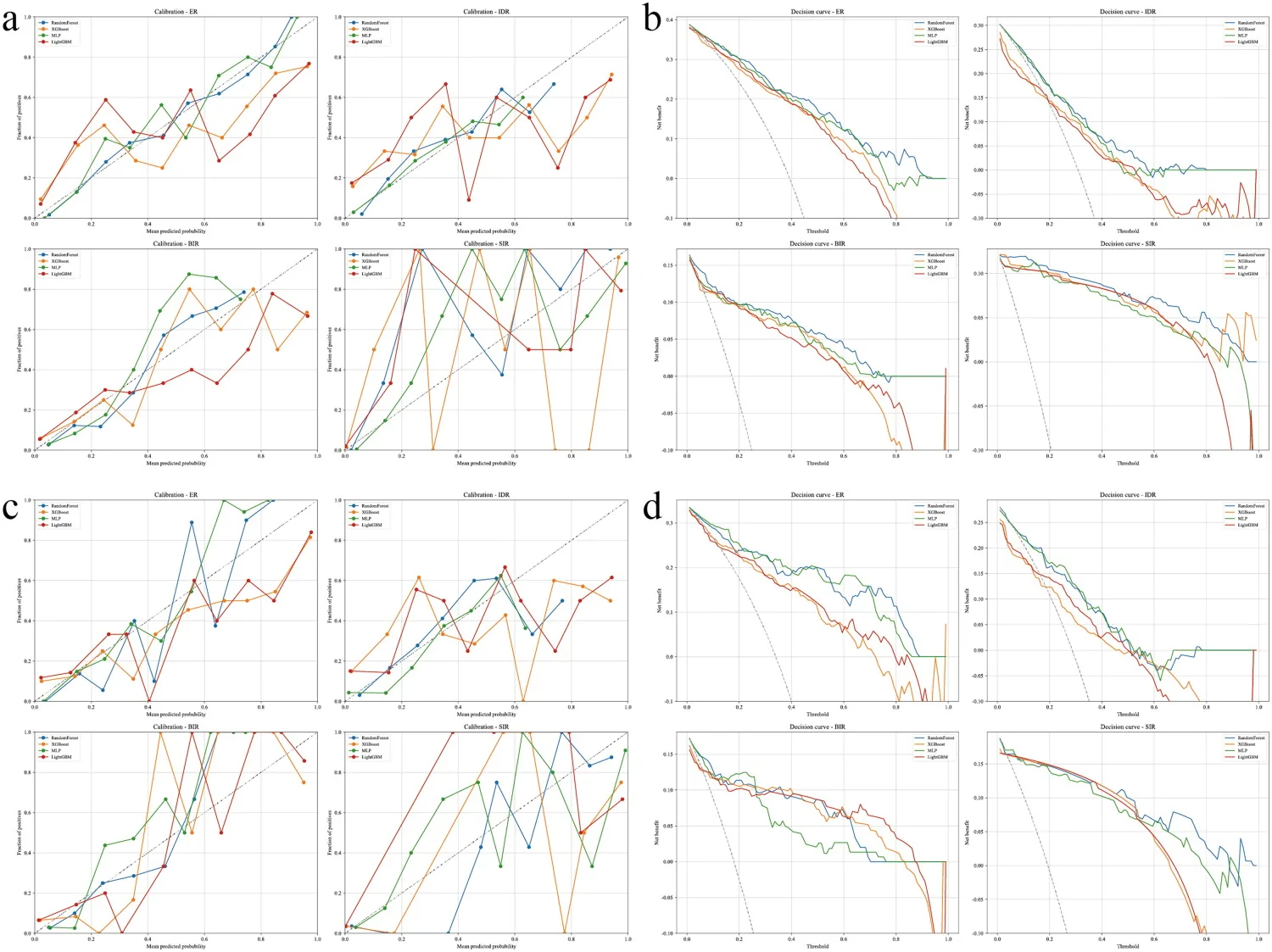
**Calibration curve and decision curve analysis (DCA) of the predictive models.** The probability estimation accuracy and clinical net benefit are stratified across response categories (ER, IDR, BIR and SIR) for the four algorithms. (a) Calibration curves in the retrospective cohort and (c) the independent temporal validation cohort, demonstrating robust diagonal alignment for the Random Forest model and severe probability fluctuations for the boosting models. (b) Decision curves in the retrospective cohort and (d) the temporal validation cohort, highlighting that the Random Forest algorithm yields the highest clinical net benefit within the crucial threshold range of 0.2 to 0.8.

The calibration and decision curve analyses are illustrated in Figure 2. The calibration curves in the training cohort (Figure 2a) and independent temporal validation cohort (Figure 2c) demonstrated that the RF model exhibited optimal and robust calibration, with its predicted probabilities showing a strong overall concordance with the diagonal reference line across response categories. In contrast, XGBoost and LightGBM displayed severe fluctuations and poor calibration curves, particularly in the BIR and SIR groups, indicating substantial prediction instability. Furthermore, decision curve analysis revealed that the RF model yielded the highest net clinical benefit across a wide range of threshold probabilities in both the training cohort (Figure 2b) and validation cohort (Figure 2d), especially within the clinically relevant threshold range of 0.2 to 0.8.

### Confusion matrix and risk score distribution analysis

**Figure 3.**
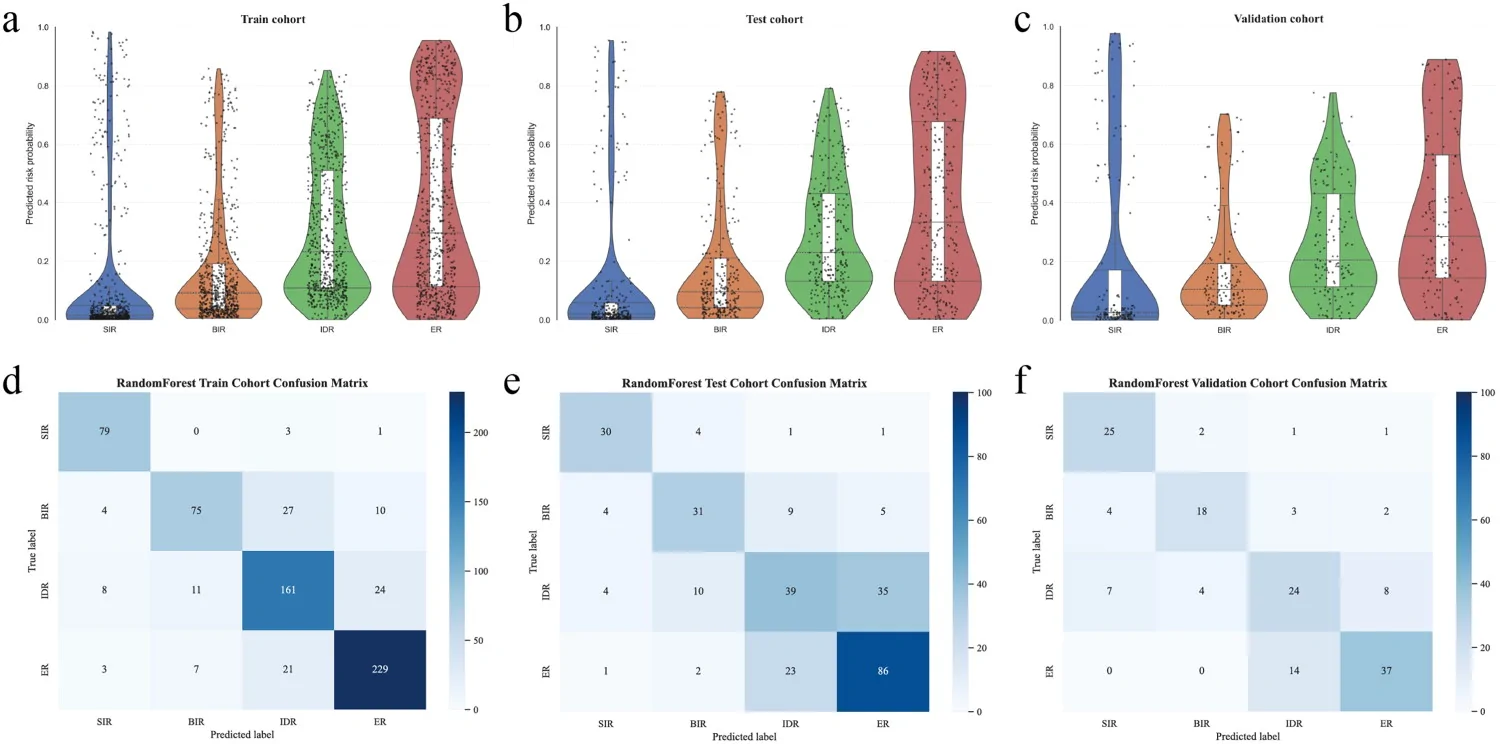
**Category-specific risk score distributions and multi-class classification accuracy.** (a–c) Violin plots illustrating the progressive, multi-tiered gradient of model-predicted risk scores (SIR < BIR < IDR < ER) across the training, test and temporal validation cohorts. The tight clustering of the SIR group (blue) near the baseline indicates high phenotypic certainty. (d–f) Confusion matrix heatmaps of the Random Forest model for the training, test and validation sets, respectively. Rows represent true clinical outcomes and columns represent predicted categories, showing a distinct diagonal dominance and minimal cross-classification beyond adjacent transitional states.

Violin plots and confusion matrix heatmaps for the four response categories are shown in Figure 3. The violin plots (Figure **3a–3c****)** demonstrated a distinct, progressive gradient in predicted probabilities across categories (SIR < BIR < IDR < ER) that remained highly consistent across all cohorts. Notably, the SIR group (blue) clustered densely near the 0.0 baseline with minimal variance, indicating exceptional classification certainty for this high-risk phenotype.

The confusion matrices (Figure **3d–3f**) confirmed strong diagonal dominance. The RF model correctly classified 79, 75, 161 and 229 cases in the training set (Figure **3d**); 30, 31, 39 and 86 cases in the test set (Figure **3e**); and 25, 18, 24 and 37 cases in the validation set for SIR, BIR, IDR and ER, respectively **(**Figure **3f**). Aligning with the high certainty shown in the violin plots, the SIR category achieved near-perfect isolation with minimal errors. Misclassifications were primarily restricted to adjacent categories (e.g. IDR and ER/BIR), confirming the model’s stable and precise four-class stratification capability.

### SHAP analysis reveals category-specific feature drivers

**Figure 4.**
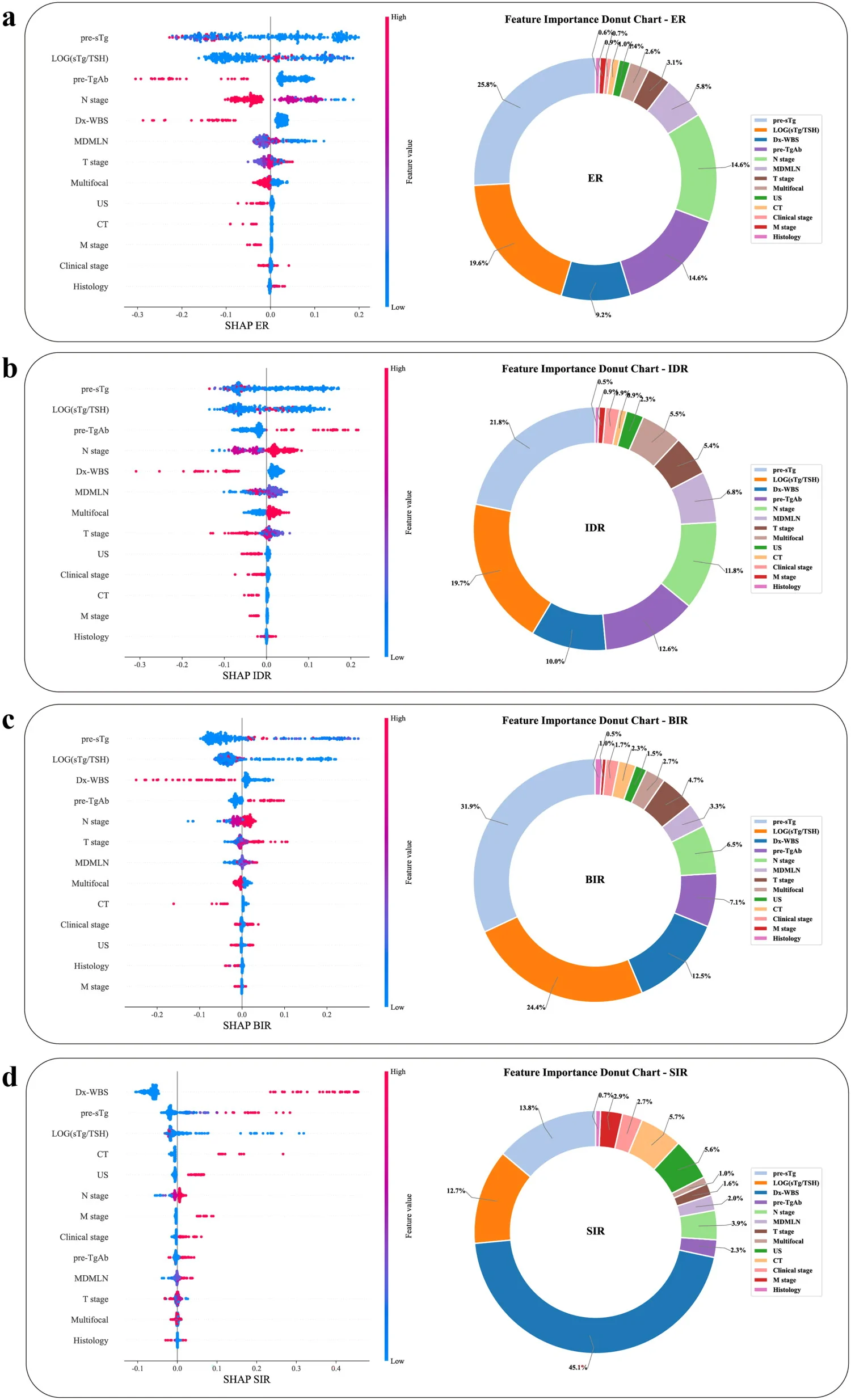
**Interpretability analysis of category-specific clinical drivers** *via* **SHAP weights.** Beeswarm plots (left) visualize the directional impact of individual feature values (red indicating high value, blue indicating low value) on specific outcomes, while donut charts (right) quantify the corresponding mean absolute SHAP importance percentages. The feature impact profiles are decoupled into: (a) Excellent Response (ER), dominated by low baseline biomarkers; (b) Indeterminate Response (IDR), characterized by clinical ambiguity; (c) Biochemical Incomplete Response (BIR), driven primarily by pre-sTg elevation and (d) Structural Incomplete Response (SIR), definitively dominated by Dx-WBS findings. **Abbreviations:** Dx-WBS: diagnostic whole-body scan; LOG(sTg/TSH): logarithmically transformed stimulated thyroglobulin-to-thyrotropin ratio; MDMLN: maximum diameter of metastatic lymph node; pre-sTg: pre-ablation stimulated thyroglobulin; pre-TgAb: pre-ablation anti-thyroglobulin antibody; SHAP: SHapley Additive exPlanations; TNM: tumor-node-metastasis.

The employment of SHAP analysis yielded the identification of category-specific drivers (Figure 4). In the ER group, pre-sTg (25.8%), LOG(sTg/TSH) (19.6%) and pre-TgAb (14.6%) were the predominant features, and low values of these biomarkers were indicative of ER. In the IDR group, pre-sTg (21.8%) and LOG(sTg/TSH) (19.7%) exhibited a near-zero value, indicative of the clinical ambiguity of an indeterminate response. In the BIR group, pre-sTg exerted its strongest influence (31.9%), with high values driving BIR. In the SIR group, Dx-WBS exhibited a dominant prevalence of 45.1%, with distant metastasis demonstrating a strong predictive capacity. Cross-category comparison confirmed distinct mechanisms: pre-sTg importance ranged from 31.9% (BIR) to 13.8% (SIR), while Dx-WBS surged from 12.5% (BIR) to 45.1% (SIR), confirming that biochemical and structural incomplete responses were driven by different factors.

### Association of key features with efficacy outcomes

**Figure 5.**
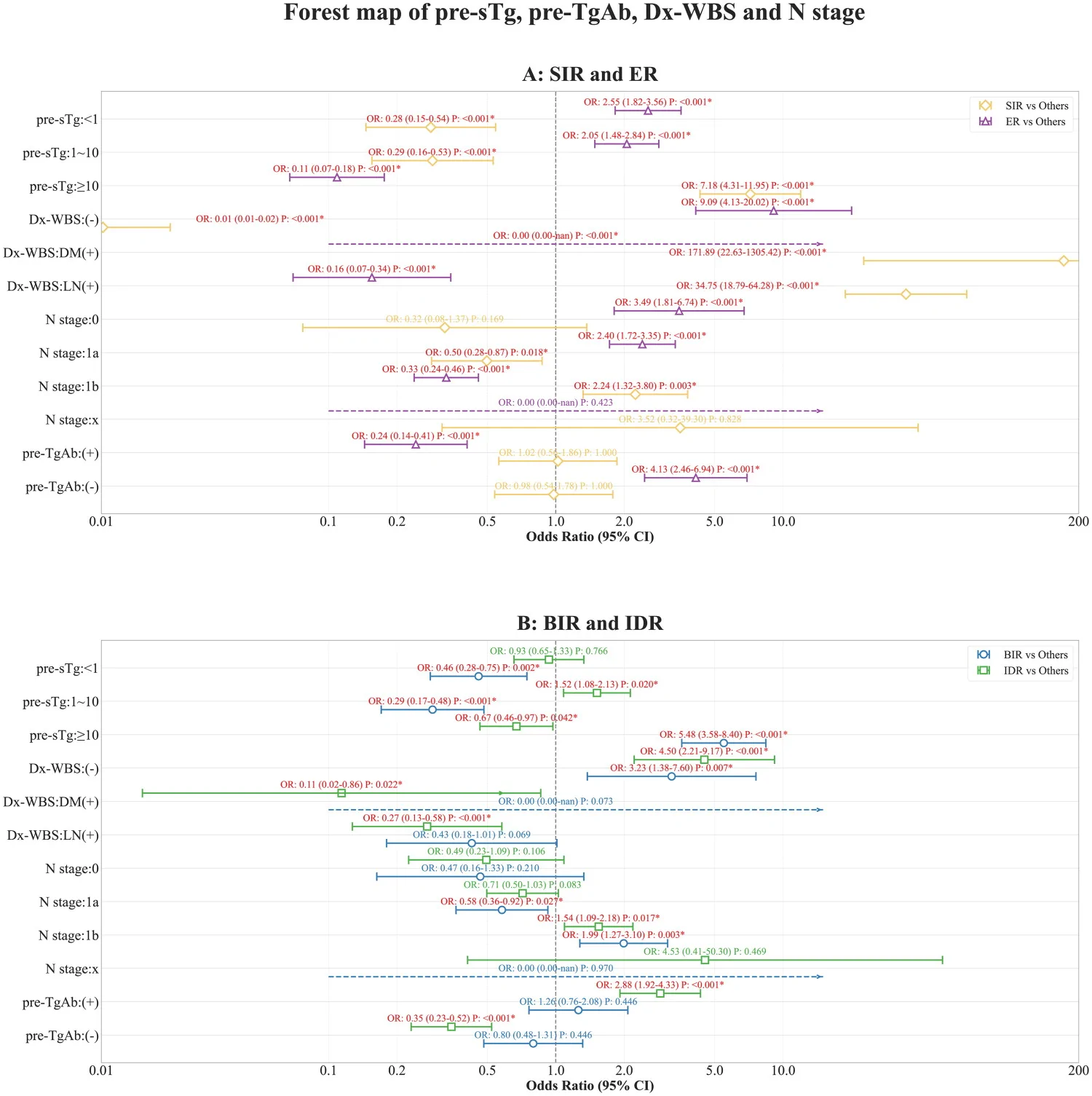
**Multivariable logistic regression forest plots delineating outcome-specific odds ratios.** Adjusted odds ratios (ORs) and 95% confidence intervals (CIs) quantify the independent predictive strengths of SHAP-prioritized features across One-vs-Rest binary comparison frameworks. (A) Multivariable analysis comparing SIR vs. Others (confirming pre-sTg ≥ 10 ng/mL and positive Dx-WBS as major risk factors) and ER vs. Others (confirming negative Dx-WBS as a strong predictor for remission, while pre-sTg ≥ 10 ng/mL heavily reduces ER odds). (B) Multivariable analysis comparing BIR vs. Others and IDR vs. Others response models. In each model, the target outcome is compared against the pooled combination of the other three cohorts (e.g. ER vs. IDR+BIR+SIR) to evaluate the independent effect of each clinical covariate subgroup (reference categories: pre-sTg < 1 ng/mL, Dx-WBS(-), N0, and pre-TgAb(-)). **Abbreviations:** CI: confidence interval; DM: distant metastasis; Dx-WBS: diagnostic whole-body scan; LOG(sTg/TSH): logarithmically transformed stimulated thyroglobulin-to-thyrotropin ratio; MDMLN: maximum diameter o.f metastatic lymph node; OR: odds ratio; pre-sTg: pre-ablation stimulated thyroglobulin; pre-TgAb: pre-ablation anti-thyroglobulin antibody; TR: thyroid bed remnant. **Statistical annotations:** Asterisks indicate statistical significance (* *P* < 0.05).

Multivariable logistic regression identified independent predictors using One-vs-Rest binary comparison frameworks (Figure 5). For the SIR vs. Others model (Figure 5A), Dx-WBS distant metastasis (DM) and lymph node (LN) metastasis dramatically elevated SIR risk, with adjusted ORs of 171.89 (95% CI: 22.63–1305.42) and 34.75 (95% CI: 18.79–64.28), respectively. Additionally, a pre-sTg ≥10 ng/mL significantly increased SIR risk (OR: 7.18, 95% CI: 4.31–11.95). Concurrently, for the ER vs. Others model, a negative Dx-WBS finding (OR: 9.09, 95% CI: 4.13–20.02) independently predicted higher odds of achieving ER, whereas an elevated pre-sTg ≥10 ng/mL strongly reduced the odds of achieving an ER outcome (OR: 0.11, 95% CI: 0.07–0.18).

For the BIR and IDR models (Figure 5B), the primary metabolic driver for BIR was pre-sTg ≥10 ng/mL (OR: 5.48, 95% CI: 3.58–8.40). Dx-WBS findings exerted a limited impact, with only cervical LN metastasis weakly correlating with BIR (OR: 3.23). Concurrently, positive pre-TgAb(+) status independently predicted a higher likelihood of entering the IDR state (OR: 2.88, 95% CI: 1.92–4.33).

## Discussion

This study constructed and independently validated a novel, highly interpretable four-class machine learning prediction system to delineate post-operative I-131 therapy responses (ER, IDR, BIR and SIR) in patients with differentiated thyroid carcinoma (DTC). The primary clinical and scientific contribution of this work lies in the rejection of traditional binary efficacy classification, which oversimplifies the therapeutic spectrum, and the subsequent implementation of a multi-class framework capable of decoupling category-specific driving mechanisms *via* SHAP analysis [12]. By advancing predictive oncology from a rudimentary binary judgment to a nuanced, multi-dimensional assessment of efficacy outcomes, this framework provides robust, evidence-based support for personalized, pre-treatment risk stratification [6,7]. In clinical practice, this 4-class model serves as a dynamic companion to baseline ATA risk categories by tailoring surveillance and dosing for intermediate-risk patients before therapy.

A major methodological and clinical innovation of this study is the elegant dual-verification framework that bridges algorithmic non-linear feature mining (SHAP) with rigorous clinical quantification *via* multivariable logistic regression forest plots (Figure 5) [13]. Unlike traditional binary models that compress dependencies into a singular axis [12], our multivariable forest plots effectively translate category-specific vectors into adjusted odds ratios (ORs) with precise 95% confidence intervals (CIs). This framework successfully unmasks the contrasting pathophysiological drivers governing extreme efficacy outcomes. In the SIR and ER evaluation models (Figure 5A), when evaluated against the ultra-low tumour-burden baseline, a pre-sTg level ≥ 10 ng/mL manifests a clear divergence. It independently propels patients towards structural progression with a robust adjusted OR of 7.18 in the SIR model, while severely hindering the probability of achieving a complete cure in the ER model, yielding an adjusted OR of 0.11 (95% CI: 0.07–0.18) [14].

Conversely, its predictive weight in the ER cohort is heavily complemented by diagnostic whole-body scintigraphy (Dx-WBS) findings. Crucially, the forest plots delineated that a negative Dx-WBS finding dramatically escalates the likelihood of achieving an excellent response, with a commanding multivariable-adjusted OR of 9.09 (95% CI: 4.13–20.02), whereas distant metastasis (DM) on Dx-WBS serves as the definitive driving engine for structural persistence (OR: 171.89) [15]. This critical divergence proves that while an elevated pre-sTg acts as a general alarm for persistent tumour ballast, the true clinical realization of a structural incomplete response is definitively dictated by macrometastatic anatomical lesions [16]. By identifying and quantitatively validating these distinct category-specific engines, our model empowers clinicians to dissect the functional–anatomical essence of persistent disease prior to therapy.

Crucially, the complex clinical reality of this multi-tiered response spectrum is mathematically validated by the newly integrated visualization tools – the violin plots and confusion matrix heatmaps (Figure 3). The violin plots (Figures 3a–3c) unveil a distinct, progressive risk probability gradient (SIR < BIR < IDR < ER) that demonstrates remarkable topological stability across the training, test and temporal validation cohorts. Notably, the high-risk SIR phenotype clusters densely and tightly near the baseline with minimal variance, proving that the model achieves near-absolute certainty when segregating the structural persistence phenotype [17]. Conversely, the confusion matrices (Figures 3d–3f) present a robust diagonal dominance while revealing a calculated degree of boundary fuzziness exclusively confined to adjacent states, particularly between indeterminate response (IDR) and its neighbouring cohorts (ER and BIR). Importantly, the complete absence of cross-contamination between distant outcomes (e.g. zero misclassification between ER and SIR) mathematically confirms our core hypothesis: post-I-131 response is inherently a continuous biological spectrum rather than a set of discrete, isolated compartments [18].

From a pathological perspective, IDR represents a highly volatile transitional ‘grey zone’ wherein patients either undergo delayed, slow glandular ablation or harbour early, covert biochemical relapse heavily masked by potential anti-thyroglobulin antibody (TgAb) interference [19]. This biological ambiguity is perfectly mirrored by the SHAP beeswarm profiles (Figure 4b), where the core feature vectors for the IDR cohort tightly cluster around the zero baseline, indicating a lack of absolute, dominant driving signals. Our multivariable forest plots (Figure 5B) harmoniously resonated with this clinical entanglement, showing that positive pre-TgAb was significantly associated with a higher likelihood of IDR (OR: 2.88, 95% CI: 1.92–4.33), further unmasking the antibody-driven masking effect that complicates IDR stratification [20]. Consequently, the true clinical utility of our framework shifts from a rigid categorical assignment to the evaluation of the continuous predicted probability spectrum derived from the violin plots **(Figures 3a–c)**. An IDR patient whose probability profile skews heavily towards BIR warrants intensified 3-month biochemical surveillance and early neck ultrasound, whereas an IDR patient skewing towards ER (independently predicted by negative pre-TgAb with an OR of 4.13, Figure 5A) can be safely managed with standard active surveillance, thereby bridging the gap between algorithmic labels and dynamic patient triage [21,22].

The superior generalizability and robust calibration demonstrated by the RF model over gradient boosting algorithms (XGBoost and LightGBM) are intimately tied to the mathematical nature of clinical oncology data. While XGBoost and LightGBM achieved absolute mathematical saturation (AUC = 1.000) on the training set, they suffered a performance decline (0.13 to 0.15 drop in AUC) in the randomized test cohort, unmasking severe overfitting [23,24]. Clinical registries are inherently plagued by measurement variation, potential noise and relatively small sample sizes (*N* = 948) [25]. By constructing an ensemble of independent decision trees *via* bagging and feature subspace sampling, the RF model effectively dampened noise propagation and mitigated overfitting, sustaining optimal micro-average AUCs of 0.894 and 0.885 in the test and independent validation cohorts, respectively [26]. Decision curve analysis (DCA) further confirmed that this algorithmic stability translates into a superior net clinical benefit across the critical threshold range of 0.2 to 0.8, cementing its real-world clinical practicality.

Despite these strengths, several limitations inherent to this study provide clear pathways for future multi-modal evolution. First, the single-centre retrospective design introduces potential selection bias. While the independent temporal validation cohort demonstrated excellent external generalizability, prospective multi-centre trials across distinct regional standards are warranted to confirm global reproducibility [27]. Second, molecular profiles (e.g. BRAF V600E and TERT promoter mutations) were not available in our retrospective cohort; future prospective multi-centre studies should evaluate whether incorporating longitudinal genomic markers can further sharpen the discriminatory boundary between IDR and BIR/SIR [28]. Given that molecular dedifferentiation directly impairs iodine avidity and shifts tumours towards SIR phenotypes, the omission of these markers may underestimate risks in molecularly high-risk subsets. Future iterations should transition towards a multi-modal fusion framework that integrates clinical parameters with high-dimensional radiomics features and single-cell multi-omics. Such deep integration will undoubtedly sharpen the classification boundaries of the chaotic IDR transitional state and pave the way for a comprehensive, closed-loop clinical decision support system (CDSS) [29]. Third, wide confidence intervals observed in certain subgroups in multivariable analysis** (**Figure 5**) **reflect event sparsity in rare clinical sub-categories, which warrants validation in larger prospective cohorts.

In conclusion, this study establishes a novel, highly interpretable four-class machine learning prediction system that successfully decodes the dynamic biochemical and structural drivers of I-131 therapy responses in DTC. As an advanced, dynamic supplement to standard AJCC staging and ATA risk stratification, this framework transitions endocrine oncology from rigid, standardized care towards a highly granular, precision medicine paradigm.

## Supplementary Material

Supplementary Table 1.docx

Study protocol.docx

Supplementary Table 2.docx

Table_2 clean file.docx

Ethics Approval Letter.pdf

Table_1 (1) clean file.docx

## Data Availability

The data sets generated and/or analysed during the current study are not publicly available due to patient privacy and ethical considerations, but are available from the corresponding author upon reasonable request.
