# Supplementary material for: Interpretable four-class machine learning prediction of initial I-131 therapy responses in differentiated thyroid cancer
Source: Ann Med. 2026 Sep 10;58(1):2729596. doi: 10.1080/07853890.2026.2729596 (PMC13564170; doi:10.1080/07853890.2026.2729596)
Supplement: Supplementary file 1 [file IANN_A_2729596_SM8299.pdf]

南昌大学第一附属医院医学伦理委员会审查意见函

审查意见号：IIT【2026】临伦审第 435 号

**项目名称**	预测甲状腺癌患者对 1311 治疗的异质性反应：一种结合 SHAP 驱动特征洞察的四分类机器学习方法		
**资助方/主持单位**	南昌大学第一附属医院		
**研究类别**	研究者发起的研究		
**专业组**	核医学科	**主要研究者**	张庆
**审查时间**	2026-04-15	**审查地点**	/
**审查形式**	快速审查	**审查类别**	初始审查
**审查文件**			
研究方案(版本号:第 1.0 版，版本日期:2026 年 03 月 12 日)；免除知情同意审查申请表； 主要研究者履历； 主要研究者声明； 专业组项目研究团队说明；			
**审查意见**	**1、批准**<br>**2、跟踪审查频率 12 个月/次，请于 2027 年 04 月 15 日 前一个月递交跟踪审查**		
注意事项	1、研究实施前提：所有研究实施过程中应符合赫尔辛基宣言和 GCP 的基本原则。如涉及人类遗传资源保护的项目，获得中国人类遗传资源办公室批准/备案后，将批准/备案成功文件递交给伦理委员会备案后方可实施。<br>2、研究过程中对研究方案和知情同意书等资料有任何修改请提交伦理委员会审查同意后方可实施。<br>3、请按南昌大学第一附属医院伦理委员会要求递交相关安全性报告。<br>4、方案违背/偏离需及时上报本伦理委员会。<br>5、根据伦理委员会对跟踪审查频度的意见，无论试验开始与否，请于跟踪审查到期前一个月提出跟踪审查的申请。<br>6、暂停/终止临床研究请及时通知本伦理委员会。<br>7、研究结束时，需提交结题报告给伦理委员会。		

主任委员签字：

日期：2026-04-15

南昌大学第一附属医院医学伦理委员会（盖章）

声明：本伦理委员会严格按照 GCP，ICH-GCP 及相关法规组成和工作

地址：南昌市永外正街 17 号 联系人：舒展、郑颖 电话&传真：0791-88692201

IITS2026419 南昌大学第一附属医院医学伦理委员会
