# Supplementary material for: Interpretable four-class machine learning prediction of initial I-131 therapy responses in differentiated thyroid cancer
Source: Ann Med. 2026 Sep 10;58(1):2729596. doi: 10.1080/07853890.2026.2729596 (PMC13564170; doi:10.1080/07853890.2026.2729596)
Supplement: Supplementary file 2 [file IANN_A_2729596_SM8298.docx]

**Table 1.** Distribution of Baseline Characteristics of the Study Population

| **Clinical Data** | | **Total**  **(N=948)** | **ER** | **IDR** | **BIR** | **SIR** |
| --- | --- | --- | --- | --- | --- | --- |
| Efficacy (%) | ER | 372(39.2) |  |  |  |  |
|  | IDR | 292(30.8) |  |  |  |  |
|  | BIR | 165(17.4) |  |  |  |  |
|  | SIR | 119(12.6) |  |  |  |  |
| Gender | Male | 293 (30.9) | 106(28.5) | 93(31.8) | 53(32.1) | 41(34.4) |
|  | Female | 655 (69.1) | 266(71.5) | 199(68.2) | 112(67.9) | 78(65.6) |
| ^I-131^ Therapy Interval | <3 months | 641 (67.6) | 270(72.8) | 193(65.7) | 102(61.8) | 76 (63.9) |
|  | >3 months | 307 (32.4) | 102(27.2) | 99(34.3) | 63(38.2) | 43 (36.1) |
| Tumor Unilateral/Bilateral | Unilateral | 420 (44.3) | 159(42.9) | 111(38.5) | 101(60.1) | 49 (42.9) |
|  | Bilateral | 528 (55.7) | 213(57.1) | 181(61.5) | 64(39.9) | 70 (57.1) |
| Extrathyroidal Invasion | Yes | 422 (44.4) | 160(43.0) | 121(41.4) | 80(48.5) | 61(51.3) |
|  | No | 526 (55.6) | 212(57.0) | 171(58.6) | 85(51.5) | 58(48.7) |
| Tumor Focality | Single | 402 (42.4) | 177(47.6) | 113(38.7) | 67(40.6) | 45(37.8) |
|  | Multiple | 546 (57.6) | 195(52.4) | 179(61.3) | 98(59.4) | 74(62.2) |
| Lateral Cervical LN Metastasis | Yes | 536 (55.3) | 155(41.7) | 184(63.0) | 112(67.9) | 85(71.4) |
|  | No | 412 (44.7) | 217(58.3) | 108(37.0) | 53(32.1) | 34(28.6) |
| Histology | Classic | 849 (89.6) | 332(89.2) | 268(91.7) | 156(94.5) | 93(78.1) |
|  | Non-classic | 77 (8.1) | 35(9.5) | 24(8.3) | 6(3.6) | 12(10.1) |
|  | Follicular carcinoma | 22 (2.3) | 5(1.3) | 0(0) | 3(1.9) | 14(11.8) |
| TgAb | Negative | 800 (84.4) | 347(93.3) | 216(74.0) | 138(83.6) | 99(83.2) |
|  | Positive | 148 (15.6) | 25(6.7) | 76(26.0) | 27(16.4) | 20(16.8) |
| T Stage | TX | 41 (4.3) | 11(3.0) | 8(2.7) | 6(3.6) | 16(13.4) |
|  | T1a | 155 (16.5) | 80(21.5) | 52(17.8) | 16(9.6) | 7(5.9) |
|  | T1b | 178 (18.8) | 75(20.2) | 62(21.2) | 28(17.0) | 13(10.9) |
|  | T2 | 128 (13.5) | 43(11.5) | 36(12.3) | 30(18.1) | 19(16.0) |
|  | T3a | 25 (2.6) | 3(0.8) | 13(4.5) | 5(3.0) | 4(3.4) |
|  | T3b | 294 (31.0) | 110(29.6) | 93(31.8) | 49(30.0) | 42(35.3) |
|  | T4a | 120 (12.7) | 48(12.9) | 26(8.9) | 30(18.1) | 16(13.4) |
|  | T4b | 7 (0.7) | 2(0.5) | 2(0.8) | 1(0.6) | 2(1.7) |
| N Stage | NX | 6 (0.6) | 0(0) | 2(0.7) | 1(0.6) | 3(2.5) |
|  | N0 | 66 (7.0) | 40(10.8) | 11(3.8) | 7(4.2) | 8(6.7) |
|  | N1a | 323 (34.1) | 170(45.7) | 87(29.8) | 42(25.5) | 24(20.2) |
|  | N1b | 553 (58.3) | 162(43.5) | 192(65.7) | 115(69.7) | 84(70.6) |
| M Stage | MX | 9 (0.9) | 0(0) | 0(0) | 4(2.4) | 5(4.2) |
|  | M0 | 911 (96.1) | 372(100) | 290(99.3) | 160(97.0) | 89(74.8) |
|  | M1 | 28 (3.0) | 0(0) | 2(0.7) | 1(0.6) | 25(21.0) |
| Clinical Stage | I | 791 (83.4) | 319(85.8) | 262(89.7) | 142(86.1) | 68(57.1) |
|  | II | 111 (11.7) | 39(10.5) | 25(8.6) | 15(9.1) | 32(26.9) |
|  | III | 29 (3.1) | 14(3.7) | 4(1.4) | 8(4.8) | 3(2.5) |
|  | IVA | 3 (0.3) | 0(0) | 1(0.3) | 0(0) | 2(1.7) |

**Table 1 (Continued)**

| **Clinical Data** | | **Total**  **(N=948)** | **ER** | **IDR** | **BIR** | **SIR** |
| --- | --- | --- | --- | --- | --- | --- |
|  | IVB | 14 (1.5) | 0(0) | 0(0) | 0(0) | 14(11.8) |
| Recurrence Risk Stratification | Low-risk | 128 (13.5) | 78(21.0) | 36(12.3) | 10(6.1) | 4(3.4) |
|  | Intermediate-risk | 566 (59.7) | 260(69.9) | 206(70.5) | 71(43.0) | 29(24.4) |
|  | High-risk | 254 (26.8) | 34(9.1) | 50(17.2) | 84(50.9) | 86(72.3) |
| US Findings | No metastasis | 836 (88.2) | 349(93.8) | 267(91.4) | 141(85.5) | 79(66.4) |
|  | LN metastasis | 112 (11.8) | 23(6.2) | 25(8.6) | 24(14.5) | 40(33.6) |
| CT Findings | No metastasis | 916 (96.6) | 372(100.0) | 289(99.0) | 165(100.0) | 90(75.6) |
|  | Metastasis | 32 (3.4) | 0(0) | 3(1.0) | 0(0) | 29(24.4) |
| Dx-WBS Findings | No metastasis | 824 (86.9) | 364(97.8) | 279(95.5) | 157(95.2) | 24(20.2) |
|  | LN metastasis | 94 (9.9) | 8(2.2) | 10(3.5) | 8(4.8) | 68(57.1) |
|  | DM | 30 (3.2) | 0(0) | 3(1.0) | 0(0) | 27(22.7) |
| Age (years) |  | 40(31.8, 50) | 43(33.5, 51) | 37 (31, 47) | 37(28, 47) | 47(32, 58.5) |
| Tumor Size(cm) |  | 1.6(1.1,2.5) | 1.5(1.0, 2.0) | 1.6(1.1, 2.2) | 1.8(1.3, 2.5) | 2.0(1.5, 3.1) |
| MDMLN(cm) |  | 0.9(0.6,1.1) | 0.8(0.4, 1.0) | 0.9(0.6, 1.1) | 1.0(0.8, 1.2) | 1.0(0.8, 1.4) |
| pre-TSH (mIU/L) |  | 82.1(61.1,100) | 79.6(60.6, 100) | 82.6(61.6, 100) | 85.0(62.2, 100) | 79.1(58.1, 100) |
| pre-sTg (ng/mL) |  | 4.2(0.6, 15.9) | 1.6(0.3, 4.1) | 4.5(0.3, 10.5) | 25.1(8.9, 58.2) | 78.4(10.5, 406.0) |
| sTg/TSH ratio |  | 0.05(0.01, 0.2) | 0.02(0.01, 0.05) | 0.06 (0.01, 0.14) | 0.3(0.1, 0.7) | 0.9(0.2, 5.7) |
| Weight (kg) |  | 61.5(55,70) | 61(55, 70) | 61(55, 70) | 62(55, 71) | 62.5(54.8, 70) |
| Height (cm) |  | 160(157,167) | 160(157, 165) | 161(157,168) | 160(156, 168) | 160(157, 167.5) |
| BMI (kg/m²) |  | 23.8(21.5,26.1) | 23.9 (21.7, 26.4) | 23.8(21.3, 25.7) | 24.1 (21.4, 26.3) | 23.6 (21.6, 25.6) |
| I-131 Dose MBq (mCi) |  | 5550(150)  [ 5550, 6660 (150,180)] | 5550(150)  [5550, 5550  (150, 150)] | 5550(150)  [5550, 5920 (150,160)] | 6660(180)  [5550, 6660  (150, 180)] | 6660(180)  [5920, 7400  (160, 200)] |

* Data are presented as n (%) or median (IQR) unless otherwise indicated.

**Abbreviations:** ER, excellent response; IDR, indeterminate response; BIR, biochemical incomplete response; SIR, structural incomplete response; IQR, interquartile range; ATA, American Thyroid Association; pre-TgAb, pre-treatment thyroglobulin antibody; pre-sTg, pre-treatment stimulated thyroglobulin; pre-TSH, pre-treatment thyroid-stimulating hormone; sTg/TSH ratio, stimulated thyroglobulin-to-thyroid-stimulating hormone ratio; Dx-WBS, diagnostic whole-body scintigraphy; MDMLN, maximum diameter of metastatic lymph node; LN, lymph node; DM, distant metastasis; US, ultrasound; CT, computed tomography; BMI, body mass index; TNM, tumor-node-metastasis; MBq, megabecquerel; mCi, millicurie.

**Note:** In accordance with journal guidelines, I-131 therapeutic doses are simultaneously expressed in both Megabecquerels (MBq) as the primary unit and Millicuries (mCi) in parentheses: 5550 MBq (150 mCi), 5920 MBq (160 mCi), 6660 MBq (180 mCi), and 7400 MBq (200 mCi). Diagnostic whole-body scintigraphy (Dx-WBS) was performed with a diagnostic dose of 222 MBq (6 mCi) of I-131 prior to therapeutic administration. **Dx-WBS findings were visually evaluated by two nuclear medicine physicians, with additional SPECT/CT performed for suspicious focal lesions to categorize uptake into Dx-WBS(-), Dx-WBS:LN(+), and Dx-WBS:DM(+). Serum pre-sTg was assessed across all patients, with pre-TgAb status incorporated as an independent covariate to account for potential antibody immunoassay interference during downstream modeling.**
