# Supplementary material for: Interpretable four-class machine learning prediction of initial I-131 therapy responses in differentiated thyroid cancer
Source: Ann Med. 2026 Sep 10;58(1):2729596. doi: 10.1080/07853890.2026.2729596 (PMC13564170; doi:10.1080/07853890.2026.2729596)
Supplement: Supplementary file 3 [file IANN_A_2729596_SM8300.docx]

**Table 2.** Univariate Analysis of Clinical Features Across ER, IDR, BIR, and SIR Groups

| **Feature** | ***χ²*** | ***p*** | **Feature** | ***Kruskal-H*** | ***p*** |
| --- | --- | --- | --- | --- | --- |
| Gender | 1.95 | 0.580 | Age | 36.42 | <0.001 |
| Therapy Interval | 7.79 | 0.050 | Tumor Size | 51.84 | <0.001 |
| Tumor Unilateral/Bilateral | 1.75 | 0.630 | MDMLN | 26.15 | <0.001 |
| Tumor Focality | 21.11 | <0.005 | pre-TSH | 6.26 | 0.100 |
| Extrathyroidal Invasion | 4.70 | 0.190 | pre-sTg | 276.77 | <0.001 |
| Lateral Cervical LN Metastasis | 57.84 | <0.001 | sTg/TSH ratio | 262.20 | <0.001 |
| Histology | 61.98 | <0.001 | Weight | 0.48 | 0.920 |
| pre-TgAb | 46.57 | <0.001 | Height | 2.53 | 0.470 |
| T Stage | 74.11 | <0.001 | BMI | 1.07 | 0.780 |
| N Stage | 72.88 | <0.001 | I-131 Dose | 219.46 | <0.001 |
| M Stage | 180.37 | <0.001 |  |  |  |
| Clinical Stage | 150.36 | <0.001 |  |  |  |
| Recurrence Risk Stratification | 257.40 | <0.001 |  |  |  |
| US Findings | 76.94 | <0.001 |  |  |  |
| CT Findings | 169.90 | <0.001 |  |  |  |
| Dx-WBS Findings | 541.44 | <0.001 |  |  |  |

**Abbreviations:** pre-TgAb, pre-treatment thyroglobulin antibody; pre-sTg, pre-treatment stimulated thyroglobulin; pre-TSH, pre-treatment thyroid-stimulating hormone; sTg/TSH ratio, stimulated thyroglobulin-to-thyroid-stimulating hormone ratio; US, ultrasound; CT, computed tomography; Dx-WBS, diagnostic whole-body scintigraphy; MDMLN, maximum diameter of metastatic lymph node; BMI, body mass index; TNM, tumor-node-metastasis; *χ^2^*, chi-square test statistic; H / Kruskal-H, Kruskal-Wallis H test statistic.
