# Supplementary material for: Interpretable four-class machine learning prediction of initial I-131 therapy responses in differentiated thyroid cancer
Source: Ann Med. 2026 Sep 10;58(1):2729596. doi: 10.1080/07853890.2026.2729596 (PMC13564170; doi:10.1080/07853890.2026.2729596)
Supplement: Supplementary file 4 [file IANN_A_2729596_SM8301.docx]

**Supplementary Table 2. Class-specific and overall predictive performance of four machine learning models across training, test, and temporal validation cohorts.**

|  |  |  | **Train**  **cohort** |  |  |  |  |
| --- | --- | --- | --- | --- | --- | --- | --- |
|  |  | AUC | 95%CI | Sensitivity | Specificity | Accuracy | F1 |
| **RF** | ER | 0.965 | 0.952-0.975 | 0.881 | 0.913 | 0.900 | 0.869 |
|  | IDR | 0.949 | 0.933-0.963 | 0.789 | 0.889 | 0.858 | 0.773 |
|  | BIR | 0.971 | 0.958-0.982 | 0.647 | 0.967 | 0.911 | 0.712 |
|  | SIR | 0.997 | 0.995-0.999 | 0.952 | 0.974 | 0.971 | 0.893 |
|  | microAUC | 0.971 | 0.962-0.978 | 0.817 | 0.936 | 0.821 | 0.817 |
|  | macroAUC | 0.971 | 0.963-0.977 | 0.817 | 0.936 | 0.821 | 0.812 |
| **XGBoost** | ER | 0.861 | 0.818-0.900 | 0.768 | 0.763 | 0.765 | 0.996 |
|  | IDR | 0.753 | 0.696-0.806 | 0.443 | 0.832 | 0.712 | 0.993 |
|  | BIR | 0.891 | 0.840-0.932 | 0.633 | 0.932 | 0.881 | 0.996 |
|  | SIR | 0.988 | 0.978-0.996 | 0.833 | 0.964 | 0.947 | 1.000 |
|  | microAUC | 1.000 | 0.991-1.000 | 0.997 | 0.998 | 0.995 | 0.995 |
|  | macroAUC | 1.000 | 1.000-1.000 | 0.997 | 0.998 | 0.995 | 0.996 |
| **MLP** | ER | 0.870 | 0.840-0.896 | 0.773 | 0.799 | 0.789 | 0.658 |
|  | IDR | 0.803 | 0.767-0.837 | 0.642 | 0.758 | 0.722 | 0.450 |
|  | BIR | 0.830 | 0.789-0.872 | 0.422 | 0.958 | 0.864 | 0.017 |
|  | SIR | 0.974 | 0.960-0.986 | 0.663 | 0.979 | 0.940 | 0.470 |
|  | microAUC | 0.886 | 0.848-0.888 | 0.625 | 0.874 | 0.658 | 0.505 |
|  | macroAUC | 0.870 | 0.850-0.899 | 0.625 | 0.874 | 0.658 | 0.399 |
| **LightGBM** | ER | 1.000 | 1.000-1.000 | 0.988 | 1.000 | 0.995 | 0.703 |
|  | IDR | 1.000 | 1.000-1.000 | 0.995 | 0.991 | 0.992 | 0.451 |
|  | BIR | 1.000 | 1.000-1.000 | 0.991 | 0.998 | 0.997 | 0.604 |
|  | SIR | 1.000 | 1.000-1.000 | 1.000 | 1.000 | 1.000 | 0.811 |
|  | microAUC | 1.000 | 1.000-1.000 | 0.994 | 0.997 | 0.992 | 0.628 |
|  | macroAUC | 1.000 | 1.000-1.000 | 0.994 | 0.997 | 0.992 | 0.642 |

|  |  |  | **Test**  **cohort** |  |  |  |  |
| --- | --- | --- | --- | --- | --- | --- | --- |
|  |  | AUC | 95%CI | Sensitivity | Specificity | Accuracy | F1 |
| **RF** | ER | 0.861 | 0.818-0.900 | 0.768 | 0.763 | 0.765 | 0.800 |
|  | IDR | 0.753 | 0.696-0.806 | 0.443 | 0.832 | 0.712 | 0.653 |
|  | BIR | 0.891 | 0.850-0.932 | 0.633 | 0.932 | 0.881 | 0.515 |
|  | SIR | 0.988 | 0.987-0.996 | 0.833 | 0.964 | 0.947 | 0.726 |
|  | microAUC | 0.894 | 0.842-0.901 | 0.669 | 0.873 | 0.653 | 0.663 |
|  | macroAUC | 0.875 | 0.845-0.900 | 0.669 | 0.873 | 0.653 | 0.673 |
| **XGBoost** | ER | 0.827 | 0.775-0.871 | 0.750 | 0.746 | 0.747 | 0.686 |
|  | IDR | 0.706 | 0.642-0.707 | 0.432 | 0.807 | 0.961 | 0.448 |
|  | BIR | 0.862 | 0.808-0.912 | 0.592 | 0.932 | 0.874 | 0.583 |
|  | SIR | 0.983 | 0.969-0.994 | 0.778 | 0.968 | 0.944 | 0.771 |
|  | microAUC | 0.867 | 0.813-0.874 | 0.638 | 0.863 | 0.628 | 0.611 |
|  | macroAUC | 0.874 | 0.813-0.875 | 0.638 | 0.863 | 0.628 | 0.622 |
| **MLP** | ER | 0.843 | 0.797-0.887 | 0.714 | 0.780 | 0.754 | 0.733 |
|  | IDR | 0.723 | 0.666-0.779 | 0.591 | 0.741 | 0.695 | 0.452 |
|  | BIR | 0.861 | 0.786-0.926 | 0.571 | 0.970 | 0.902 | 0.612 |
|  | SIR | 0.972 | 0.943-0.990 | 0.694 | 0.984 | 0.947 | 0.538 |
|  | microAUC | 0.876 | 0.817-0.881 | 0.643 | 0.689 | 0.649 | 0.558 |
|  | macroAUC | 0.852 | 0.817-0.880 | 0.643 | 0.689 | 0.649 | 0.431 |
| **LightGBM** | ER | 0.843 | 0.785-0.878 | 0.741 | 0.757 | 0.751 | 0.703 |
|  | IDR | 0.700 | 0.636-0.763 | 0.420 | 0.817 | 0.695 | 0.451 |
|  | BIR | 0.860 | 0.801-0.912 | 0.592 | 0.911 | 0.856 | 0.604 |
|  | SIR | 0.979 | 0.963-0.992 | 0.806 | 0.968 | 0.947 | 0.811 |
|  | microAUC | 0.866 | 0.812-0.872 | 0.640 | 0.863 | 0.626 | 0.628 |
|  | macroAUC | 0.845 | 0.810-0.872 | 0.640 | 0.863 | 0.626 | 0.642 |

|  |  |  | **Validation cohort** |  |  |  |  |
| --- | --- | --- | --- | --- | --- | --- | --- |
|  |  | AUC | 95%CI | Sensitivity | Specificity | Accuracy | F1 |
| **RF** | ER | 0.895 | 0.840-0.945 | 0.725 | 0.889 | 0.883 | 0.667 |
|  | IDR | 0.774 | 0.686-0.849 | 0.558 | 0.832 | 0.753 | 0.400 |
|  | BIR | 0.903 | 0.837-0.961 | 0.667 | 0.591 | 0.900 | 0.565 |
|  | SIR | 0.924 | 0.861-0.976 | 0.862 | 0.909 | 0.900 | 0.704 |
|  | microAUC | 0.885 | 0.832-0.912 | 0.703 | 0.95 | 0.693 | 0.593 |
|  | macroAUC | 0.878 | 0.828-0.910 | 0.703 | 0.95 | 0.693 | 0.584 |
| **XGBoost** | ER | 0.841 | 0.770-0.902 | 0.765 | 0.768 | 0.767 | 0.631 |
|  | IDR | 0.724 | 0.637-0.804 | 0.349 | 0.850 | 0.707 | 0.148 |
|  | BIR | 0.871 | 0.781-0.952 | 0.630 | 0.967 | 0.907 | 0.303 |
|  | SIR | 0.931 | 0.878-0.974 | 0.862 | 0.909 | 0.900 | 0.750 |
|  | microAUC | 0.851 | 0.799-0.885 | 0.651 | 0.874 | 0.640 | 0.532 |
|  | macroAUC | 0.864 | 0.794-0.885 | 0.651 | 0.874 | 0.640 | 0.458 |
| **MLP** | ER | 0.910 | 0.857-0.952 | 0.725 | 0.848 | 0.807 | 0.121 |
|  | IDR | 0.776 | 0.694-0.851 | 0.651 | 0.738 | 0.713 | 0.138 |
|  | BIR | 0.878 | 0.799-0.941 | 0.370 | 0.959 | 0.853 | 0.056 |
|  | SIR | 0.972 | 0.943-0.990 | 0.694 | 0.984 | 0.947 | 0.542 |
|  | microAUC | 0.882 | 0.828-0.909 | 0.609 | 0.872 | 0.633 | 0.193 |
|  | macroAUC | 0.876 | 0.829-0.911 | 0.609 | 0.872 | 0.633 | 0.179 |
| **LightGBM** | ER | 0.834 | 0.785-0.878 | 0.741 | 0.757 | 0.751 | 0.688 |
|  | IDR | 0.700 | 0.636-0.763 | 0.420 | 0.817 | 0.695 | 0.078 |
|  | BIR | 0.860 | 0.801-0.912 | 0.952 | 0.911 | 0.856 | 0.242 |
|  | SIR | 0.979 | 0.963-0.992 | 0.806 | 0.968 | 0.947 | 0.591 |
|  | microAUC | 0.851 | 0.781-0.879 | 0.654 | 0.875 | 0.640 | 0.507 |
|  | macroAUC | 0.837 | 0.785-0.877 | 0.654 | 0.875 | 0.640 | 0.400 |
