# Supplementary material for: Interpretable four-class machine learning prediction of initial I-131 therapy responses in differentiated thyroid cancer
Source: Ann Med. 2026 Sep 10;58(1):2729596. doi: 10.1080/07853890.2026.2729596 (PMC13564170; doi:10.1080/07853890.2026.2729596)
Supplement: Supplementary file 5 [file IANN_A_2729596_SM8302.docx]

**CLINICAL STUDY PROTOCOL**

**Project Title:** Interpretable Four-Class Machine Learning Prediction of Initial I-131 Therapy Responses in Differentiated Thyroid Cancer

**Protocol Version:** Version 1.0

**Protocol Date:** March 12, 2026

**Study Institution:** The First Affiliated Hospital, Jiangxi Medical College, Nanchang University

**Principal Investigator (PI):** Qing Zhang, MD, PhD (Department of Nuclear Medicine)

**Sponsor:** The First Affiliated Hospital, Jiangxi Medical College, Nanchang University

**1. BACKGROUND AND RATIONALE**

Differentiated thyroid carcinoma (DTC) is the most common malignant endocrine neoplasm, with a rising global incidence. Post-operative radioiodine (I-131) therapy reduces recurrence risk in intermediate- and high-risk patients. However, therapeutic responses vary substantially, ranging from Excellent Response (ER), Biochemical Incomplete Response (BIR), Indeterminate Response (IDR), to Structural Incomplete Response (SIR).

Conventional risk prediction relies heavily on static pathological features (e.g., TNM staging and ATA risk stratification). These methods lack sensitivity for dynamic therapeutic responses. Machine learning (ML) models offer strengths in integrating multi-dimensional clinical data. However, existing studies are limited by two primary bottlenecks: (1) relying on oversimplified binary classifications ("excellent" vs. "non-excellent") that obscure transitional states like IDR and BIR; and (2) lacking algorithmic interpretability.

To overcome these gaps, this study establishes and validates an interpretable four-class machine learning framework. By combining Shapley Additive exPlanations (SHAP) with multivariable statistical modeling, we aim to decouple the outcome-specific clinical drivers governing post-I-131 responses.

**2. STUDY OBJECTIVES**

1) **Primary Objective:** To construct, optimize, and externally validate a four-class machine learning prediction system (ER, IDR, BIR, SIR) for initial I-131 therapy in DTC patients using routinely collected clinical data.
2) **Secondary Objectives:**
a) To identify outcome-specific drivers across different response categories using SHAP analysis.
b) To quantify the independent predictive strengths of key predictors for incomplete outcomes (BIR and SIR) via multivariable logistic regression.
c) To provide a continuous probability profile for dynamic clinical risk stratification and patient management.

**3. STUDY DESIGN AND METHODOLOGY**

**Study Type:** Single-center, retrospective observational study with an independent temporal validation cohort. (Compliant with STROBE guidelines).

**Study Setting:** Departments of Nuclear Medicine, Ultrasound, and Thyroid Surgery, The First Affiliated Hospital of Nanchang University.

1) **Study Population & Timeline:**
a) **Development Cohort (Retrospective):** 948 DTC patients treated with initial I-131 therapy between October 2020 and March 2024 (randomly split into 70% training [n=663] and 30% test [n=285] sets).
b) **Independent Temporal Validation Cohort:** 150 non-overlapping DTC patients treated between October 2024 and March 2025.
c) **Follow-up Duration:** All patients were followed up for $\geq6$months post-I-131 therapy to confirm treatment response.
2) **Eligibility Criteria**
a) **Inclusion Criteria:**
i. Histologically confirmed DTC (papillary or follicular thyroid carcinoma).
ii. Total or near-total thyroidectomy followed by initial I-131 ablation/therapy.
iii. Complete baseline clinical, laboratory, imaging, and 6-month follow-up data.
b) **Exclusion Criteria:**
i. Non-differentiated thyroid carcinomas (e.g., medullary or anaplastic carcinoma).
ii. Prior non-I-131 oncological treatments (e.g., radiotherapy, chemotherapy, or targeted therapy).
iii. Concurrent malignancies or severe systemic organ dysfunction.
iv. Incomplete data or loss to follow-up.

**4. VARIABLES AND DATA COLLECTION**

Routinely collected clinical predictors (26 initial variables) were extracted from electronic health records:

1) **Demographics:** Age, sex, BMI.
2) **Histopathology:** T/N/M stage, clinical stage, pathological subtype, multifocality, extrathyroidal extension.
3) **Laboratory Parameters:** Pre-ablation stimulated thyroglobulin (pre-sTg), pre-TSH, LOG(sTg/TSH) ratio, pre-TgAb status.
4) **Imaging Findings:** Thyroid ultrasound, chest CT, diagnostic whole-body scintigraphy (Dx-WBS).
5) **Primary Outcome:** 6-month post-I-131 therapeutic response categorized into ER, IDR, BIR, or SIR according to the 2025 ATA guidelines.

**5. STATISTICAL ANALYSIS AND MACHINE LEARNING PIPELINE**

1) **Feature Selection:** Univariate analysis followed by Variance Inflation Factor (VIF) filtering (threshold VIF $<10$) to eliminate multicollinearity, resulting in 13 core predictors.
2) **Model Training:** Four algorithms (Random Forest, XGBoost, LightGBM, and Multi-Layer Perceptron) were built using 5-fold nested cross-validation on the training set.
3) **Performance Assessment:** Evaluated on the test (n=285) and temporal validation (n=150) cohorts using micro/macro-average AUCs, calibration curves, and decision curve analysis (DCA).
4) **Interpretability & Forest Plots:** Global and local feature importances were quantified using SHAP values. Multivariable logistic regression forest plots were constructed to derive adjusted Odds Ratios (ORs) and 95% CIs.

**6. ETHICAL CONSIDERATIONS AND DATA MANAGEMENT**

1) **Ethical Approval:** The protocol was reviewed and approved by the Ethics Committee of The First Affiliated Hospital of Nanchang University (Approval No. IIT [2026] Clinical Ethics Review No. 435).
2) **Informed Consent Waiver:** Due to the retrospective nature of the medical registry analysis, requirement for individual informed consent was waived by the Ethics Committee.
3) **Data Privacy:** All patient health records were de-identified and anonymized prior to statistical analysis in strict compliance with the Declaration of Helsinki.
4) **Timeline of Data Analysis:** Electronic medical records and clinical data were formally extracted between April 18, 2026 and June 5, 2026, following formal ethical approval, after which machine learning modeling and statistical analyses were performed.

**Principal Investigator Signature:** Qing Zhang, MD, PhD

**Date:** March 12, 2026
