# Supplementary material for: Interpretable four-class machine learning prediction of initial I-131 therapy responses in differentiated thyroid cancer
Source: Ann Med. 2026 Sep 10;58(1):2729596. doi: 10.1080/07853890.2026.2729596 (PMC13564170; doi:10.1080/07853890.2026.2729596)
Supplement: Supplementary file 6 [file IANN_A_2729596_SM8303.docx]

**Supplementary Table 1.** Baseline Clinical Features Stratified by Dataset Partitioning

| **Clinical Data** | | **Total**  **(N=948)** | **Training Set**  **(N=663)** | **Test Set**  **(N=285)** | **Validation Set**  **(N=150)** |
| --- | --- | --- | --- | --- | --- |
| Efficacy (%) | ER | 372(39.2) | 260(39.2) | 112(39.3) | 51(34.0) |
|  | IDR | 292(30.8) | 204(30.8) | 88(30.9) | 43(28.7) |
|  | BIR | 165(17.4) | 116(17.5) | 49(17.2) | 29(19.3) |
|  | SIR | 119(12.6) | 83(12.5) | 36(12.6) | 27(18.0) |
| Gender | Male | 293 (30.9) | 198 (29.9) | 95 (33.3) | 54 (36.0) |
|  | Female | 655 (69.1) | 465 (70.1) | 190 (66.7) | 96 (64.0) |
| ^I-131^ Therapy Interval | <3 months | 641 (67.6) | 440 (66.4) | 201 (70.5) | 111 (74.0) |
|  | >3 months | 307 (32.4) | 223 (33.6) | 84 (29.5) | 39 (26.0) |
| Tumor Unilateral/Bilateral | Unilateral | 420 (44.3) | 295 (44.5) | 125 (43.9) | 48 (32.0) |
|  | Bilateral | 528 (55.7) | 368 (55.5) | 160 (56.1) | 102 (68.0) |
| Extrathyroidal Invasion | Yes | 422 (44.4) | 299 (45.1) | 123 (43.2) | 88(58.7) |
|  | No | 526 (55.6) | 364 (54.9) | 162 (56.8) | 62(41.3) |
| Tumor Focality | Single | 402 (42.4) | 293(44.2) | 109 (38.2) | 68(45.3) |
|  | Multiple | 546 (57.6) | 370 (55.8) | 176 (61.8) | 82(54.7) |
| Lateral Cervical LN Metastasis | Yes | 536 (55.3) | 382 (57.6) | 154 (54.0) | 120(70.0) |
|  | No | 412 (44.7) | 281 (42.4) | 131 (46.0) | 30(20.0) |
| Histology | Classic | 849 (89.6) | 602 (90.8) | 247 (86.7) | 116 (77.3) |
|  | Non-classic | 77 (8.1) | 50 (7.5) | 27 (9.5) | 26 (17.3) |
|  | Follicular carcinoma | 22 (2.3) | 11 (1.7) | 11 (3.8) | 8 (5.4) |
| TgAb | Negative | 800 (84.4) | 545 (82.2) | 255 (89.5) | 132 (88.0) |
|  | Positive | 148 (15.6) | 118 (17.8) | 30 (10.5) | 18 (12.0) |
| T Stage | TX | 41 (4.3) | 26 (3.9) | 15 (5.3) | 3 (2.0) |
|  | T1a | 155 (16.5) | 106 (16.0) | 49 (17.2) | 21 (14.0) |
|  | T1b | 178 (18.8) | 130 (19.6) | 48 (16.8) | 23 (15.3) |
|  | T2 | 128 (13.5) | 83 (12.5) | 45 (15.8) | 28 (18.7) |
|  | T3a | 25 (2.6) | 20 (3.0) | 5 (1.7) | 3 (2.0) |
|  | T3b | 294 (31.0) | 208 (31.4) | 86 (30.2) | 41 (27.3) |
|  | T4a | 120 (12.7) | 84 (12.7) | 36 (12.6) | 30 (20.0) |
|  | T4b | 7 (0.7) | 6 (0.9) | 1 (0.4) | 1 (0.7) |
| N Stage | NX | 6 (0.6) | 3 (0.5) | 3 (1.0) | 2 (1.3) |
|  | N0 | 66 (7.0) | 43 (6.5) | 23 (8.1) | 4 (2.7) |
|  | N1a | 323 (34.1) | 215 (32.4) | 108 (37.9) | 25 (16.7) |
|  | N1b | 553 (58.3) | 402 (60.6) | 151 (53.0) | 119 (79.3) |
| M Stage | MX | 9 (0.9) | 7 (1.1) | 2 (0.7) | 0(0) |
|  | M0 | 911 (96.1) | 640 (96.5) | 271 (95.1) | 136 (90.7) |
|  | M1 | 28 (3.0) | 16 (2.4) | 12 (4.2) | 14 (9.3) |
| Clinical Stage | I | 791 (83.4) | 561 (84.6) | 230 (80.7) | 125 (83.3) |
|  | II | 111 (11.7) | 70 (10.5) | 41 (14.4) | 17 (11.3) |
|  | III | 29 (3.1) | 21 (3.2) | 8 (2.8) | 3 (2.0) |
|  | IVA | 3 (0.3) | 3 (0.5) | 0(0) | 0(0) |

**Supplementary Table 1 (Continued)**

| **Clinical Data** | | **Total**  **(N=948)** | **Training Set**  **(N=663)** | **Test Set**  **(N=285)** | **Validation Set**  **(N=150)** |
| --- | --- | --- | --- | --- | --- |
|  | IVB | 14 (1.5) | 8 (1.2) | 6 (2.1) | 5 (3.4) |
| Recurrence Risk Stratification | Low-risk | 128 (13.5) | 96 (14.5) | 32 (11.2) | 11(7.3) |
|  | Intermediate-risk | 566 (59.7) | 394 (59.4) | 172 (60.4) | 81 (54.0) |
|  | High-risk | 254 (26.8) | 173 (26.1) | 81 (28.4) | 58 (38.7) |
| US Findings | No metastasis | 836 (88.2) | 592(89.3) | 244 (85.6) | 138 (92.0) |
|  | LN metastasis | 112 (11.8) | 71 (10.7) | 41 (14.4) | 12 (8.0) |
| CT Findings | No metastasis | 916 (96.6) | 641 (96.7) | 275 (96.5) | 136(90.7) |
|  | Metastasis | 32 (3.4) | 22 (3.3) | 10 (3.5) | 14 (9.3) |
| Dx-WBS Findings | No metastasis | 824 (86.9) | 575 (86.7) | 249 (87.4) | 115 (76.7) |
|  | LN metastasis | 94 (9.9) | 68 (10.3) | 26 (9.1) | 19 (12.7) |
|  | DM | 30 (3.2) | 20 (3.0) | 10 (3.5) | 16 (10.6) |
| Age (years) |  | 40(31.8, 50) | 40 (31, 49) | 42(32, 52) | 40(31, 49) |
| Tumor Size(cm) |  | 1.6(1.1,2.5) | 1.5(1.1,2.5) | 1.5(1.1, 2.4) | 2.0 (1.2, 2.7) |
| MDMLN(cm) |  | 0.9(0.6,1.1) | 0.8(0.6,1.1) | 0.8 (0.6, 0.9) | 1.0(0.6, 1.1) |
| pre-TSH (mIU/L) |  | 82.1(61.1,100) | 82.3 (62.1, 100) | 76.5(57.6, 100) | 84.7 (59.8, 100) |
| pre-sTg (ng/mL) |  | 4.2(0.6, 15.9) | 3.7(0.4, 15.1) | 5.3(1.3, 18.1) | 7.1 (1.7, 32.3) |
| sTg/TSH ratio |  | 0.05(0.01, 0.2) | 0.04(0, 0.2) | 0.08(0.02, 0.28) | 0.10 (0.02, 0.37) |
| Weight (kg) |  | 61.5(55,70) | 62(55,70) | 62(55, 70) | 64 (54.3, 74) |
| Height (cm) |  | 160(157,167) | 160(157,166) | 160 (157, 168) | 160 (157, 167) |
| BMI (kg/m²) |  | 23.8(21.5,26.1) | 23.8(21.5,26.3) | 23.8 (22.1, 26) | 24.8(21.7, 27) |
| I-131 Dose (mCi) |  | 5550(150)  [ 5550, 6660 (150,180)] | 5550(150)  [5550, 6660  (150, 180)] | 5550(150)  [5550, 6660  (150, 180)] | 5020(160)  [5550, 6660  (150, 180)] |

* Data are presented as n (%) or median (IQR) unless otherwise indicated. **Abbreviations:** ER, excellent response; IDR, indeterminate response; BIR, biochemical incomplete response; SIR, structural incomplete response; IQR, interquartile range; ATA, American Thyroid Association; pre-TgAb, pre-treatment thyroglobulin antibody; pre-sTg, pre-treatment stimulated thyroglobulin; pre-TSH, pre-treatment thyroid-stimulating hormone; sTg/TSH ratio, stimulated thyroglobulin-to-thyroid-stimulating hormone ratio; Dx-WBS, diagnostic whole-body scintigraphy; MDMLN, maximum diameter of metastatic lymph node; LN, lymph node; DM, distant metastasis; US, ultrasound; CT, computed tomography; BMI, body mass index; TNM, tumor-node-metastasis; MBq, megabecquerel; mCi, millicurie. Note: In accordance with journal guidelines, I-131 therapeutic doses are simultaneously expressed in both Megabecquerels (MBq) as the primary unit and Millicuries (mCi) in parentheses. Diagnostic whole-body scintigraphy (Dx-WBS) was performed with a diagnostic dose of 222 MBq (6 mCi) of I-131 prior to therapeutic administration.
